# A Novel Approach to Improving Squat Jump Performance: The Pre-Loaded Squat Jumps

**DOI:** 10.5114/jhk/200809

**Published:** 2025-09-23

**Authors:** Zhengqiu Gu, Zhili Chen, Kaifang Liao, Chris Bishop, Chong Gao, Fogen Zhong, Shengji Deng, Xinxin Wang, Jianxi Wei, Yongming Li

**Affiliations:** 1 Department of Education, Pinghu Normal college of Jiaxing University, Jiaxing, China.; 2 School of Athletic Performance, Shanghai University of Sport, Shanghai, China.; 3 Department of Strength and Conditioning, Guangdong Vocational Institute of Sport, Guangzhou, China.; 4 Faculty of Science and Technology, London Sports Institute, Middlesex University, London, UK.; 5 Research Center of Military Exercise Science, Army Engineering University of PLA, Nanjing, China.; 6 China Institute of Sport Science, Beijing, China.

**Keywords:** plyometrics, stretch-shortening cycle, accentuated eccentric load, optimum power load

## Abstract

The present study aimed to explore the acute effects of a novel preloading strategy on squat jump (SJ) kinetics and kinematics from 0 to 50% body mass. Twenty-one male college athletes (mean ± SD: age = 23.29 ± 3.15 yrs, body mass = 75.50 ± 6.20 kg, body height = 178.07 ± 6.45 cm and body fat content = 13.71 ± 5.45%) completed three different jump tasks across two testing sessions: a SJ, a preloaded SJ (10–50% body mass) and a countermovement jump (CMJ). Applying preloading before the SJ resulted in significant, trivial to small increases in jump height (F_(5,15)_ = 3.76 , p = 0.01, η^2^ = 0.16). However, there was no significant effect of preloading on peak power, peak force and peak velocity. The maximum peak power (5128.28 ± 459.38 W vs. 5047.97 ± 447.67 W, p = 0.04; g = 0.17) and maximum peak force (1810.72 ± 150.35 N vs. 1775.50 ± 155.54 N, p = 0.03; g = 0.25) were reached at the load of 20% body mass, which was significantly higher than in the SJ with no preloading applied. Jump height, peak force and peak velocity in the 10–50% body mass preloading SJ tests were not significantly different from those in the CMJ. Preloading before a SJ results in meaningful improvements in jump performance, particularly in peak power. Athletes in sports requiring high jump performance can use preloading SJ strategies to enhance lower limb explosive power and jump height.

## Introduction

Vertical jump ability is a critical component for successful performance in various sports (e.g., volleyball and basketball), serving as a key indicator for evaluating and improving lower limb explosive power in athletes (Baker, 2002; Pojskic et al., 2024). Enhancing vertical jump performance has been a prevalent topic among coaches and athletes for years (Handford, 2021; Suchomel et al., 2017). The countermovement jump (CMJ) and the squat jump (SJ) are two commonly used vertical jump tasks (Loturco et al., 2024). The CMJ involves a downward movement followed by an explosive upward acceleration, while the SJ requires holding a half-squat position before a concentric-only jump. The CMJ typically results in higher jump height (JH) and power output due to the stretch-shortening cycle (SSC), showing increases of ~7 cm and 719.4 W, respectively, compared to the SJ (Bobbert et al., 1996; Earp et al., 2010; Kabacinski et al., 2022; Mackala et al., 2013). SSC efficiency is often measured by the pre-stretch augmentation index, with a smaller index indicating reduced efficiency (Kozinc et al., 2022; Van Hooren, 2017).

Accentuated eccentric loading (AEL) is a training method that incorporates loading the eccentric portion of a movement in excess of concentric prescription, enhancing performance without interrupting natural movement mechanics (Suchomel et al., 2024; Wagle et al., 2017). Previous studies have shown that AEL in the CMJ, achieved by holding weights and removing the load before the concentric phase, can enhance JH and power output by an additional 2.1 cm and 439.6 W, respectively (Sheppard et al., 2007). Training the CMJ with AEL has been shown to have superior effects on JH, power, and velocity compared to training with body mass alone (Sheppard et al., 2008). Aboodarda et al. (2013) reported that using elastic bands for AEL during the CMJ improved JH by 4 cm, relative power output by 20.1 W/kg, and relative net impulse by 0.58 N/s/kg. Applying AEL in the CMJ results in greater eccentric velocity and force, promoting stronger motor neuron stimulation and enhancing the SSC effect, potentially storing more elastic energy during the eccentric phase, which is released during the concentric phase (Sheppard et al., 2007).

Despite the SJ lacking an SSC component, preloading strategies (e.g., maintaining a squatting position with an additional load) may induce heightened pre-activation of the agonist muscles. From our prior training experience, preloading the SJ with weight plates and dropping the load before the take-off has shown better jump performance compared to the SJ alone. This preloading approach may generate greater ground reaction force (GRF) and its effectiveness could be influenced by the CMJ-SJ_diff_, providing a new technique for improving vertical jump ability and lower body power output (Baz-Valle et al., 2019). Power output varies across exercises and loads, with the optimum power load maximizing power output in a given exercise, crucial for athletic training (Loturco et al., 2021). Training with loads that optimize external mechanical power can significantly improve dynamic athletic performance (Wilson et al., 1993). However, minimal research has investigated the optimum power load for preloading the SJ, as well as the performance of preloaded SJs under varying loads. This gap in the literature may limit the practical application of preloaded SJs in training and athletic performance enhancement.

Therefore, the present study aimed to (a) investigate the differences in JH, peak power (PP), peak force (PF), and peak velocity (PV) of preloaded SJ tasks (from 0 to 50% body mass), and (b) compare these metrics between the CMJ and preloaded SJs. We hypothesized that (a) jump-related variables in preloaded SJ tasks would be maximized at moderate loads (20–30% body mass), and (b) there would be no significant differences between moderate load preloading SJ tasks and the CMJ.

## Methods

### Participants

Twenty-three physically active sport science students voluntarily participated in this study and 21 students (age = 23.29 ± 3.15 yrs, body mass = 75.50 ± 6.20 kg, body height = 178.07 ± 6.45 cm and body fat content = 13.71 ± 5.45%) completed all tests, while two students dropped out for personal reasons. Participants were recruited through recruitment posters. Sample size was estimated a priori using G*Power 3.1 with an effect size of 0.5 (based on previous research by Aboodarda et al., 2013), an alpha level of 0.05, and a statistical power of 0.80; the calculated required sample size was 6 participants. The inclusion criteria for this research were as follows: regular engagement in physical exercise, a squat 1RM equal to or exceeding 1.5 times body mass, no history of hypertension or cardiac medical issues, and no debilitating sports-related injuries that adversely affected physical activity within the preceding year. All participants were informed about the potential risks and discomforts associated with this study and provided informed consent by signing a form. The Shanghai University of Sport Ethics Committee, Shanghai, China, approved the research (protocol code: 102772023RT102; approval date: 24 October 2023).

### Design and Procedures

This study employed a randomized repeated-measure design to identify whether preloading the SJ using dumbbells would improve jump performance as the CMJ. Subjects participated in three laboratory sessions over a two-week period, which included a familiarization session, a SJ and CMJ test session, and a preloading SJ test session. There was a recovery period of at least 48 h between each test. To ensure consistency under testing conditions, participants were instructed to wear the same style of shoes and shorts during all trials. The tests were conducted at the same time of the day in a controlled laboratory environment with constant temperature and humidity. Concurrently, verbal encouragement was provided to elicit participants' exhibition of their maximal vertical jumping capacity.

### Familiarization Session

Before data collection, participants underwent a familiarization session to obtain anthropometric data and acquaint themselves with the different jump protocols employed in the study. Body height (cm) and mass (kg) were measured using a standard stadiometer and a scale, respectively. Body composition was determined through dual-energy X-ray absorptiometry (QDT-4500, Hologic, American). Participants warmed up by running on an indoor track for 10 min, with the perceived effort rated at approximately 12 on the RPE (Rating of Perceived Exertion) 6–20 scale. Then, participants performed dynamic stretching for 10 min. After the warm up, participants were instructed to place their hands on the sides of their body, executing a squat until reaching a knee joint angle at about 90° and maintaining this posture for the purpose of acclimatization. Participants then practiced different jump protocols in the following order: standard SJ tests, followed by preloaded SJ tests using dumbbells with loads ranging from 0 to 50% body mass, and finally standard CMJ tests. Trials continued until participants achieved technical proficiency in the three jump techniques, as determined by the research team.

### SJ and CMJ Test Session

The SJ and CMJ tests were conducted within a single session, starting with 10 min of running and 10 min of whole-body dynamic stretching. Then, 10 SJ and 10 CMJ trials were performed before the actual test. Participants changed into standardized footwear and shorts, and four 14 mm reflective markers were affixed to the anterior superior iliac spine (ASIS) and the posterior superior iliac spine (PSIS) on both sides during a 10-min rest interval. Afterwards, participants preformed three standard SJ and CMJ trials on two force plates (500 × 600 × 50 mm, 9260AA, Kistler, Switzerland), with a 2-min rest interval between each trial. During the standard SJ test, participants were required to stand upright on the force plates with their hands on their hips throughout the jump. Subsequently, the participant was instructed to squat to a position where the knee joint was flexed at about 90° angle and to maintain this posture for 2–3 s, followed by a maximum jump. During the standard CMJ test, participants were required to stand upright on the force plate with their hands at a position slightly above the ASIS throughout the jump as well. They were then instructed to perform a counter-movement to a position where the knee joint was flexed at about 90° angle and jump as high as possible immediately.

### Preloaded SJ Test Session

**Figure 1:**
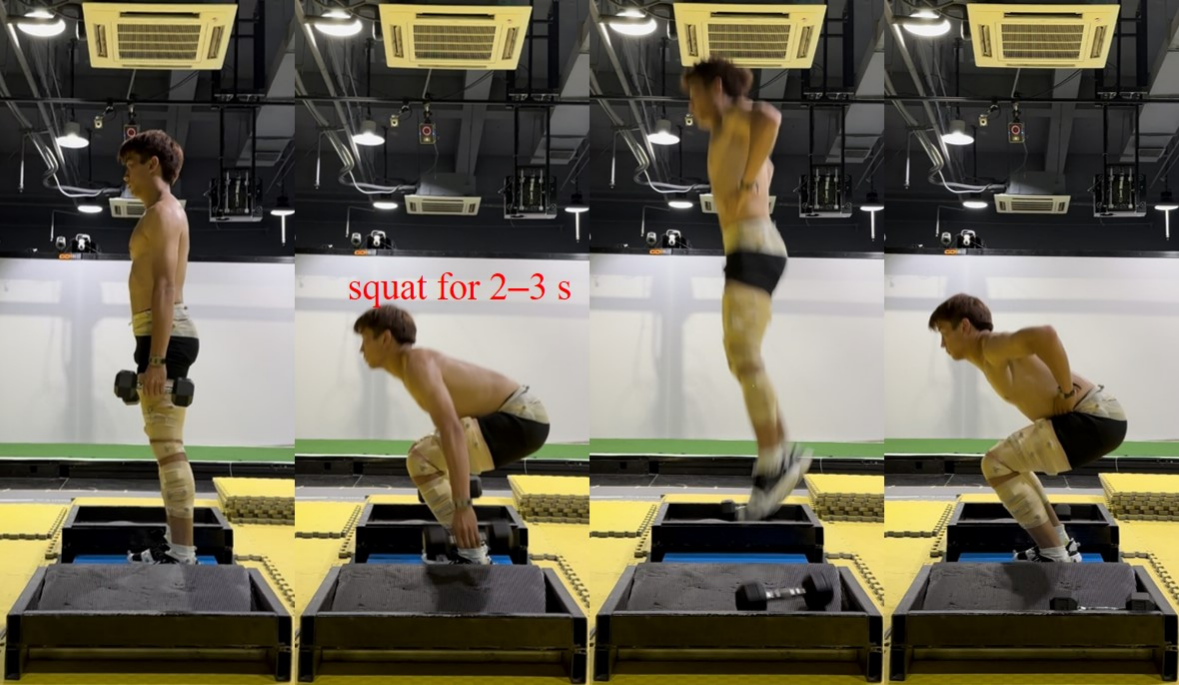
Sequence of preloading the SJ. Steel frames were outside the two force plates.

The same warm up as for the SJ and CMJ test session was executed before testing. After changing attire and markers, participants preformed a series of preloaded SJ tests with loads ranging from 10 to 50% body mass. The load conditions were randomized, and each condition comprised two trials, with the better result selected for subsequent analysis. A two-min rest interval between consecutive trials was allowed to minimize fatigue-related effects. During the preloaded SJ test, participants held a dumbbell in each hand while standing on two force plates. Subsequently, participants were instructed to squat to a position where the knee joint was flexed at about 90° angle and to maintain this posture for 2–3 s, Then, dumbbells were dropped and participants performed a maximum jump immediately. On the outside of the two force plates, steel frames were positioned to protect the force plates (Figure 1). These frames were covered with a layer of sponge padding to provide cushioning upon dumbbell release. Another function of these frames was to facilitate a natural movement when participants dropped the dumbbells.

**Figure 2:**
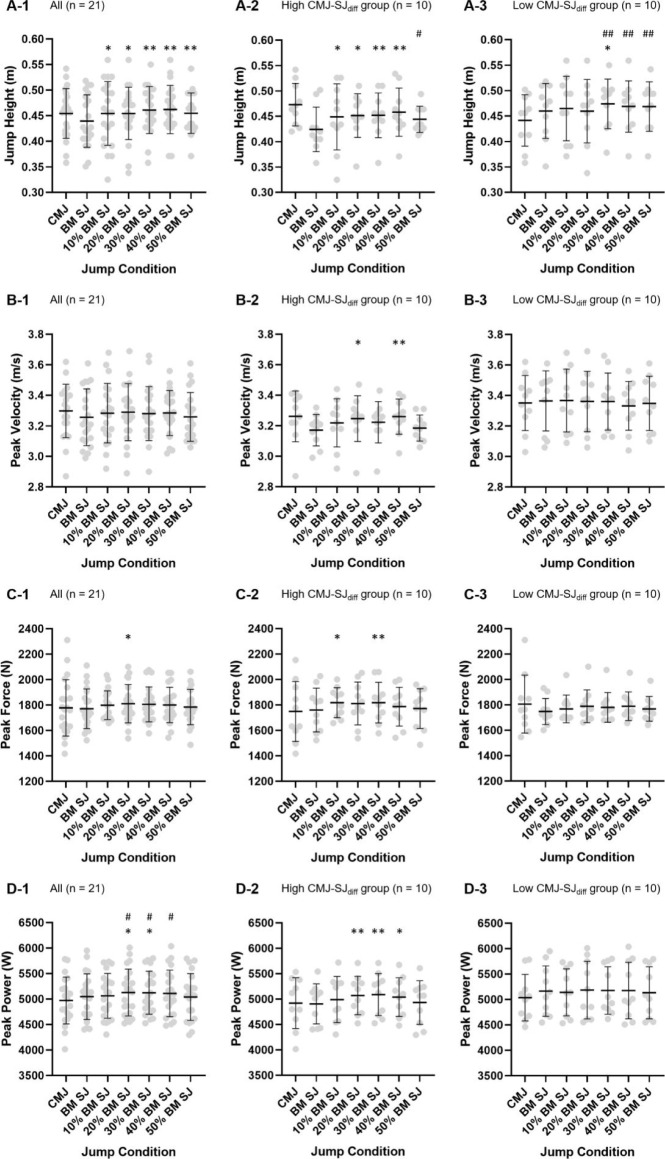
Jump height, peak power, peak force and peak velocity value in the counter-movement jump, the squat jump and the preloaded squat jump with various loads applied. * significantly different from the SJ (p < 0.05), ** significantly different from the SJ (p < 0.01), ^#^ significantly different from the CMJ (p < 0.05), ^##^ significantly different from the CMJ (p < 0.01)

The kinetic data were collected using two force plates, while kinematic data were captured by 10 optoelectronic cameras (V5, Vicon, UK) throughout all jump tests. The force plates were connected to the data processing system box and the Vantage box of Vicon for data synchronization. To address missing marker data, a gap-filling procedure was implemented utilizing rigid body methods. The sampling frequency for both kinematics and kinetics was set at 200 Hz, kinematics data were smoothed using a low-pass Butterworth filter with a cut-off frequency of 10 Hz and smooth frequency of kinetic data were 50 Hz by C-Motion Visual 3D 3.0 software. The global coordinate system was defined, where the Z-axis denoted the vertical axis, and solely the data along the vertical axis were utilized for subsequent analysis. In all jump tests, pelvic velocity was used to represent the body velocity, and the concentric phase was defined from the lowest point of the pelvis on the Z-axis to the ground reaction force (GRF) being less than 20 N. The flight time was defined as the period from the take-off GRF in the Z-axis (less than 20 N) to the landing (more than 20 N). The following variables were calculated: vertical GRF, vertical velocity, vertical power (vertical GRF × vertical velocity), jump height (calculated as 9.81 × flight time^2^/8) (Aboodarda et al., 2014).

### Statistical Analysis

Descriptive statistics were calculated and reported as means ± standard deviation. Normality of data distribution was examined by the Shapiro-Wilk test. The reliability of the jump tests was evaluated with a two-way random intraclass correlation coefficient (ICC) with 95% confidence intervals (CIs), a coefficient of variation (CV) with 95% CI and the standard error of measurement (SEM) (Table 1). To assess the effects of preloading on JH, PP, PF, and PV in the SJ across varying loads from 0% to 50% of body mass, repeated measures ANOVA was employed. The LSD post hoc tests were then used to identify specific loading conditions under which preloading significantly altered performance compared to the SJ. Paired samples *t*-tests with Hedges’ *g* effect sizes (ES), also with 95% CI, were used to compare the differences between the CMJ and each of preloaded SJ tasks. The magnitude of Hedges’ *g* ES was interpreted with the following thresholds: 0.20 < (trivial), 0.20–0.49 (small), 0.50–0.79 (moderate), ≥ 0.80 (large) (Lloyd et al., 2022). All statistical procedures were computed using SPSS software (version 27.0, SPSS, USA). The significant level was set at *p* < 0.05.

**Table 1:** Intraclass correlation coefficients for peak VGRF, peak power, velocity, and jump height for the CMJ, the SJ and the preloaded SJ.

Condition	ICC (95% CI)	CV % (95% CI)	SEM
Jump height (m)			
CMJ	0.93 (0.84, 0.97)	9.91 (5.67, 14.15)	0.01
SJ	0.96 (0.90, 0.98)	12.12 (6.93, 17.30)	0.01
10% BM Preloaded SJ	0.96 (0.91, 0.99)	13.05 (7.47, 18.63)	0.01
20% BM Preloaded SJ	0.95 (0.87, 0.98)	11.05 (6.32, 15.77)	0.01
30% BM Preloaded SJ	0.91 (0.79, 0.96)	10.01 (5.73, 14.28)	0.01
40% BM Preloaded SJ	0.96 (0.90, 0.98)	10.28 (5.88, 14.68)	0.01
50% BM Preloaded SJ	0.90 (0.77, 0.96)	9.61 (5.50, 13.72)	0.01
Peak power (W)			
CMJ	0.99 (0.96, 0.94)	9.97 (5.71, 14.24)	49.16
SJ	0.95 (0.89, 0.98)	8.77 (5.02, 12.52)	98.17
10% BM Preloaded SJ	0.95 (0.87, 0.98)	9.01 (5.16, 12.87)	101.61
20% BM Preloaded SJ	0.95 (0.88, 0.98)	8.98 (5.14, 12.82)	101.83
30% BM Preloaded SJ	0.92 (0.80, 0.97)	7.94 (4.54, 11.33)	113.68
40% BM Preloaded SJ	0.96 (0.91, 0.98)	8.64 (4.94, 12.33)	87.71
50% BM Preloaded SJ	0.96 (0.91, 0.99)	9.25 (5.29, 13.20)	92.63
Peak force (N)			
CMJ	0.98 (0.95, 1.00)	12.43 (7.11, 17.74)	31.12
SJ	0.94 (0.86, 0.98)	9.45 (5.41, 13.49)	37.10
10% BM Preloaded SJ	0.90 (0.78, 0.96)	7.99 (4.57, 11.41)	39.76
20% BM Preloaded SJ	0.96 (0.89, 0.98)	9.69 (5.55, 13.84)	31.45
30% BM Preloaded SJ	0.94 (0.85, 0.97)	8.19 (4.69, 11.70)	33.23
40% BM Preloaded SJ	0.90 (0.78, 0.96)	8.47 (4.84, 12.09)	44.93
50% BM Preloaded SJ	0.86 (0.70, 0.94)	8.59 (4.92, 12.27)	52.49
Peak velocity (m/s)			
CMJ	0.92 (0.82, 0.97)	5.10 (2.92, 7.29)	0.05
SJ peak velocity (m/s)	0.91 (0.80, 0.96)	5.78 (3.31, 8.25)	0.06
10% BM Preloaded SJ	0.94 (0.85, 0.97)	5.97 (3.42, 8.52)	0.05
20% BM Preloaded SJ	0.90 (0.78, 0.96)	5.31 (3.04, 7.59)	0.06
30% BM Preloaded SJ	0.90 (0.76, 0.96)	4.84 (2.77, 6.91)	0.05
40% BM Preloaded SJ	0.91 (0.80, 0.96)	4.98 (2.85, 7.11)	0.05
50% BM Preloaded SJ	0.91 (0.79, 0.96)	5.08 (2.91, 7.26)	0.05

Abbreviations: CMJ: countermovement jump, SJ: squat jump, CI: confidence interval, ICC: intraclass correlation coefficient, CV: coefficient of variation, SEM: standard error of measurement

**Table 2:** Hedges *g* effect sizes with 95% confidence intervals and rating between the preloaded SJ and bodyweight SJ condition.

Condition	Jump height (m)	Peak power (W)	Peak force (N)	Peak velocity (m/s)
10% BM Preloaded SJ & SJ	**0.24 (−0.39, 0.87) small**	0.03 (**−**0.59, 0.66) trivial	0.20 (**−**0.43, 0.82) small	0.14 (**−**0.49, 0.76) trivial
20% BM Preloaded SJ & SJ	**0.25 (−0.38, 0.88) small**	**0.17 (−0.45, 0.80) trivial**	**0.25 (−0.38, 0.88) small**	0.18 (**−**0.44, 0.81) trivial
30% BM Preloaded SJ & SJ	**0.42 (−0.21, 1.06) small**	**0.17 (−0.45, 0.80) trivial**	0.23 (**−**0.40, 0.86) small	0.13 (**−**0.49, 0.76) trivial
40% BM Preloaded SJ & SJ	**0.44 (−0.19, 1.07) small**	0.14 (**−**0.49, 0.76) trivial	0.20 (**−**0.43, 0.82) small	0.16 (**−**0.46, 0.79) trivial
50% BM Preloaded SJ & SJ	**0.32 (−0.31, 0.95) small**	**−**0.01 (**−**0.64, 0.61) trivial	0.09 (**−**0.54, 0.71) trivial	0.02 (**−**0.61, 0.64) trivial

Abbreviations: SJ = squat jump. Note: values in bold indicate a significant difference (p < 0.05) from the body weight SJ condition

## Results

Thirteen (61.9%) participants demonstrated a positive value for the CMJ-SJ_diff_, i.e., greater JH in the CMJ compared to the SJ. Participants were divided into two groups based on their CMJ-SJ_diff_ values. Specifically, the ten participants with the highest CMJ-SJ_diff_ values among all 21 participants were classified as the high CMJ-SJ_diff_ group, while the ten participants with the lowest CMJ-SJ_diff_ values were classified as the low CMJ-SJ_diff_ group. To maintain equal group sizes, the participant ranked 11^th^ was not assigned to either group.

### Preloaded SJ vs. SJ

In the preloaded SJ test, there was a significant effect of preloading on JH (F_(5,15)_ = 3.76 , *p* = 0.01, η^2^ = 0.16), the load of 40% body mass maximised JH (0.46 ± 0.05 m vs. 0.44 ± 0.05 m, *p* < 0.001; *g* = 0.44) across all conditions, and SJs preloaded with 10–50% body mass were all significantly higher than the SJ (*p* < 0.05; *g* = 0.24–0.44). However, there was no significant effect of preloading on PP (F_(5,15)_ = 2.34 , *p* = 0.09, η^2^ = 0.42). The maximum PP (5128.28 ± 459.38 W vs. 5047.97 ± 447.67 W, *p* = 0.04; *g* = 0.17) was achieved at the load of 20% body mass, and SJs preloaded with 20–30% body mass were significantly higher than the SJ (*p* < 0.05; *g* = 0.17). There were also no significant effect of preloading on PF (F_(5,15)_ = 1.63 , *p* = 0.18, η^2^ = 0.08) and PV (F_(5,15)_ = 0.93 , *p* = 0.45, η^2^ = 0.04). The maximum PF (1810.72 ± 150.35 N vs. 1775.50 ± 155.54 N, *p* = 0.03; *g* = 0.25) was obtained at the load of 20% body mass and was significantly higher than that of the SJ. The maximum PV (3.28 ± 0.18 m/s vs. 3.26 ± 0.19 m/s, *p* = 0.07; *g* = 0.18) was also observed at the load of 20% body mass, yet the difference was not significant when compared to the SJ.

In the high CMJ-SJ_diff_ group (CMJ-SJ_diff_ = 0.03–0.21), JH for 10–40% body mass preloaded SJs was significantly higher compared to the SJ (*p* < 0.05). Also the PP for SJs preloaded with the load of 20–40% body mass was significantly higher compared to the SJ (*p* < 0.05). Considering PF of SJs where a 10% and 30% body mass preloading strategy was applied, it was significantly higher under all preloaded conditions compared to the SJ (*p* < 0.05). With regard to PV for 20% and 40% body mass preloaded SJs, the obtained values were all significantly higher than those of the SJ (*p* < 0.05).

In the low CMJ-SJ_diff_ group (CMJ-SJ_diff_ = −0.13–0.02), only JH in 30% body mass preloaded SJs was significantly higher than in the SJ (*p* < 0.05).

### Preloaded SJ vs. CMJ

In the preloaded SJ test, PP at a load of 20–40% body mass was significantly higher than in the CMJ (*p* < 0.05; *g* = 0.30–0.33). JH (0.45 ± 0.05 m), PF (1778.31 ± 221.61 N) and PV (3.30 ± 0.18 m/s) in the 10–50% body mass preloaded SJ tests were not significantly different from those in the CMJ.

In the high CMJ-SJ_diff_ group, JH in the 50% body mass preloaded SJ was significantly lower than in the CMJ (*p* = 0.04). PP, PF and PV values in the high CMJ-SJ_diff_ group of 10–50% body mass preloaded SJs were not significantly different from those of the CMJ.

On the other hand, in the low CMJ-SJ_diff_ group, JH in 30–50% body mass preloaded SJs was significantly higher than in the CMJ (*p* < 0.01). Other values in the low CMJ-SJ_diff_ group at 10–50% body mass preloaded SJs were not significantly different from those obtained performing the CMJ.

## Discussion

The current study aimed to examine the jump performance differences among preloaded SJ tasks applying the load from 0 to 50% body mass. The primary findings indicated that implementing a preloading strategy before the SJ resulted in small, but significant increases in JH. Although there was no significant effect of preloading on PP, PF and PV, the load that maximized PP and PF was significantly higher than that observed under the SJ condition alone. JH, PF and PV in all preloaded SJ tests were not significantly different from those obtained in the CMJ. These findings support the initial hypothesis that preloading the SJ enhances jump performance, although with non-significant differences between preloaded SJ tasks and the CMJ.

The results of current study reveal that applying preloading before the take-off in a SJ is an effective method for enhancing jump performance, resulting in a JH similar to that of the CMJ. However, the improvements in performance did not continuously increase along with additional loads. The greatest improvements were observed when the load applied was low (i.e., 10% body mass), and then decreases in performance were observed when the load became larger. In addition, the maximum values for each metric did not occur at the same load. Previous research has indicated that JH during both the CMJ (4.3–10.5%) (Aboodarda et al., 2013; Sheppard et al., 2007) and the DJ (11.5%) (Aboodarda et al., 2014) can be enhanced by the addition of AEL. The present study found that preloading before a SJ can enhance JH by 5.0% at a load of 40% body mass. The proposed mechanism for this enhancement is that maintaining a squat position with an additional load during the SJ generates greater neural stimulation of the agonist muscles, resulting in supra-normal afferent nerve impulses to the central nervous system (Sheppard et al., 2007). This supra-normal input signal leads to increased efferent pulses in the extrafusal fibers, thereby enhancing jump performance. The transition from a standing to a squat position with an additional load during the SJ may elicit a greater number of actin-myosin binding sites for cross-bridge formation (Bobbert et al, 2005). Despite being maintained for only 2–3 s before the concentric phase commences, these additional cross-bridges may exert influence during the start of the concentric phase, thereby enhancing JH. That said, it should be noted that further research is needed to fully corroborate this theory.

The current study observed that, under the 20% body mass load, PF was maximized and was significantly higher than under the SJ only condition. Although the maximum PV was not significantly higher under the preloading condition, there was a trend towards higher values with ES from 0.02 to 0.18. This finding underscores the contribution of an additional load during the preloaded SJ. The squat depth requirements remained consistent under both conditions at all loads, suggesting that preloading the SJ induces higher leg stiffness than during a SJ only condition. Consequently, more elastic energy may be stored within the muscle-tendon unit and passive tendon collagen (Fukutani et al., 2017; Kemmel et al., 2018). Previous studies have found that force enhancement is observed during active lengthening, known as residual force enhancement (Seiberl et al., 2021), and can influence muscle contraction dynamics for a period as long as 20 s (De Campos et al., 2022). In the present study, jumping was performed after active lengthening for 2–3 s, suggesting the potential existence and influence of residual force enhancement on contraction dynamics. Considering the importance of power in many sports, this study found that the PP in 20% and 30% body mass preloading SJs was significantly higher than in the SJ only condition and the CMJ. Power was calculated as the product of force and velocity, and although the PF and PV in the CMJ were not significantly different from those in the preloaded SJ condition, both values presented lower trends, resulting in significantly lower PP during the CMJ when calculated thereafter. Argus et al. (2011) found that both assisted and resisted jumps using elastic bands did not improve CMJ power, suggesting that preloading the SJ could be a preferable training method for enhancing power output.

The CMJ-SJ_diff_ in the present study was 2.9 ± 9.1% (−13.1–21.3), which differs from previous research that reported a 13.0–14.0% CMJ-SJ_diff_ among physical education students (Kozinc et al., 2022). Sampling errors might be a contributing factor to the observed discrepancies. Of note, the large standard deviation and individual variance observed in our findings suggest significant fluctuations in the CMJ-SJ_diff_ within our sample, which may indicate that data should be analyzed on a more individualized basis. The group with a high CMJ-SJ_diff_ significantly improved JH at 10–40% body mass (*g* = 0.43–0.71), whereas the group with a low CMJ-SJ_diff_ significantly improved JH only at 30% body mass (*g* = 0.26), suggesting those participants were less able to benefit from the preloading strategy.

Generally, the CMJ exhibits better jump performance compared to the SJ—largely due to the SSC, with the underlying mechanisms of this phenomenon attributed to both neural and muscular factors. Preloading a SJ, which also improves jump performance, can be explained through the same aspects, suggesting a potential similarity in their mechanisms. Furthermore, the observation that the group with a high CMJ-SJ_diff_ could more effectively utilize the load before the SJ supports this hypothesis.

There are some limitations of this study which should be acknowledged. First, the selection of the load to be applied was not based on the percentage of maximum lower limb strength. However, we opted to select the load based on the percentage of participants’ body mass, taking into account individual differences. This approach was chosen due to its practicality, as it is easier to calculate and obtain the load based on a percentage of body mass. Moreover, it is a commonly used method for calculating the optimum power load. Consequently, our results are more readily generalizable. Second, the findings of the present study are based on a sample of male sport science students, which may limit the generalizability of the results to other populations, such as female athletes or sedentary individuals. Future research should investigate whether preloading can enhance the SJ performance in various populations. Additionally, future studies should examine the effectiveness of training with preloading the SJ to improve jump performance.

## Conclusions

The findings of this study indicate that preloading before a SJ results in meaningful improvements in jump performance, particularly in peak power. Athletes in sports requiring jumping performance can use preloaded SJ strategies to enhance lower limb explosive power and jump height. For athletes aiming to improve vertical jump performance, using a load of 30–50% body mass for training is recommended. However, for athletes focusing on enhancing lower limb explosive power, a training load of 20–30% body mass is suggested.
